# Effect of Physiotherapy Intervention on Improvement of Respiratory Function and Degree of Respiratory Distress Reduction in Neonates With Respiratory Distress Syndrome: Protocol for a Systematic Review

**DOI:** 10.2196/71854

**Published:** 2025-09-24

**Authors:** Sharath Hullumani, Irshad Qureshi, Raghuveer Raghumahanti, Sakshi Desai, Neha Brahmane, Pratiksha Warghat

**Affiliations:** 1 Department of Paediatrics and Neonatal Physiotherapy, Ravi Nair Physiotherapy College, Datta Meghe Institute of Higher Education and Research (DU), Sawangi Meghe, Wardha, Maharashtra, 442001, India, 91 9964066927; 2 Department of Neurophysiotherapy, Ravi Nair Physiotherapy College, Datta Meghe Institute of Higher Education and Research (DU), Wardha, Maharashtra, India

**Keywords:** NRDS, chest physiotherapy, respiration, pulmonary rehabilitation, new born, non-invasive interventions, neonatal intensive care, neonatal respiratory distress syndrome

## Abstract

**Background:**

Neonatal respiratory distress syndrome (NRDS), a common and serious illness in preterm infants, is characterized by lung immaturity and inadequate surfactant production. Even though improvements in neonatal care have increased survival rates, optimizing supportive therapies such as physiotherapy is still essential to improve respiratory outcomes and overall recovery.

**Objective:**

This study aims to evaluate the effectiveness of physiotherapy therapies in enhancing respiratory function and clinical outcomes in neonates with NRDS.

**Methods:**

PRISMA-P (Preferred Reporting Items for Systematic Reviews and Meta-Analyses Protocols) guidelines will be followed for conducting a systematic review. Studies assessing the effects of physiotherapy therapies on newborns with NRDS, such as respiratory exercises, manual chest compressions, percussion, vibration, and postural drainage, high-frequency oscillation of the chest wall, cough stimulation, or suctioning will be included. A comprehensive search of electronic databases, including PubMed, Embase, CINAHL, and the Cochrane Library, to find pertinent observational studies, experimental studies, and randomized controlled trials, will be conducted. Important outcomes will be respiratory function metrics, oxygen saturation levels, length of mechanical ventilation, occurrence of problems, and overall newborn outcomes.

**Results:**

Exact numbers are not accessible at this time, as the review is hypothetical and has not yet been conducted. In order to inform clinical procedures and future studies, the results will contain data on respiratory improvement, intervention kinds, safety profiles, and the best times and frequencies for physical therapy in neonates with respiratory distress syndrome.

**Conclusions:**

This review will provide a comprehensive understanding of the role of physiotherapy in managing NRDS and its impact on clinical outcomes in neonates. The findings may guide clinical practice and inform future research on noninvasive strategies for improving neonatal respiratory care.

## Introduction

### Rationale

Neonatal respiratory distress syndrome (NRDS) is a leading cause of morbidity and mortality among preterm infants. It primarily results from insufficient pulmonary surfactant production, which compromises alveolar stability and gas exchange. The incidence of NRDS is inversely proportional to gestational age, affecting approximately 40%‐60% of neonates born before 28 weeks of gestation and 15%‐30% of those born between 32 and 36 weeks [1]. NRDS presents significant clinical challenges, necessitating early diagnosis and prompt intervention to improve survival and reduce complications.

A portion of newborns still suffer from chronic respiratory compromise in spite of improvements in neonatal care, such as the use of prenatal corticosteroids, exogenous surfactant administration, and respiratory support techniques, such as mechanical ventilation or continuous positive airway pressure (CPAP) [2-4]. In order to maximize lung function and clinical recovery, this has led to research into supplementary therapies including physical therapy.

In the neonatal intensive care unit, physical therapy includes a variety of therapies aimed at promoting respiratory function, including positioning, vibration, chest percussion, and expiratory flow increase exercises. In newborns experiencing respiratory distress, these methods seek to improve ventilation-perfusion matching, facilitate secretion clearance, and increase oxygenation [5]. Theoretically, physiotherapy has several advantages; however, there are conflicting data regarding its effectiveness and safety in treating NRDS. According to a number of studies, physiotherapy may help newborns experiencing respiratory distress achieve better clinical outcomes, including lower oxygen requirements, improved arterial blood gases (ABGs), and shorter hospital stays. Other publications, however, express concerns about possible negative consequences, such as hypoxemia and reactions associated with stress [6]. These discrepancies could result from differences in timing, therapeutic strategies, and therapist skill.

Furthermore, the scope and methods of earlier evidence syntheses on newborn physiotherapy have been constrained, frequently concentrating on a small number of therapies or merging diverse populations without sufficient stratification. The impact of physiotherapy in newborns with NRDS especially has to be thoroughly assessed, and the relevant data must be consolidated. The purpose of this systematic review is to evaluate the safety and efficacy of physiotherapy techniques as NRDS adjunctive treatments. This review will give a thorough grasp of the available data, point out knowledge gaps, and guide future clinical guidelines for infant respiratory treatment by combining findings from observational and interventional studies.

The findings of earlier systematic reviews that looked at physiotherapy in neonates were not as applicable to neonates with NRDS since they frequently encompassed diverse populations, such as newborns with bronchiolitis, pneumonia, or postsurgical disorders. A majority of these reviews combined different respiratory conditions without classifying the results according to gestational age or diagnosis, 2 important factors in the treatment of NRDS [7,8]. Furthermore, current syntheses usually place more emphasis on short-term physiological metrics such as respiratory rate or oxygen saturation rather than clinically significant outcomes such as mortality, hospital stay, or length of respiratory support. In addition, few evaluations have addressed how to manage heterogeneity in interventions, demographics, and end measures, or examined the efficacy of various physiotherapy modalities in a systematic manner. These gaps highlight the necessity of a targeted, diagnosis-specific review of the literature regarding physiotherapy for newborns with NRDS [9].

### Objective

Assessing the effectiveness of physiotherapy therapies in improving respiratory function and lowering distress in infants with respiratory distress syndrome (RDS) is the goal of this systematic review methodology. Analyzing the decrease in respiratory support requirements, classifying the different forms and protocols of physiotherapy, and assessing the improvement in clinical respiratory scores are among the goals. The review will also look into the physiological mechanisms, safety, and viability of physiotherapy in this population, as well as the best times and frequencies of interventions to optimize therapeutic results. This thorough investigation will help to improve neonatal care for patients with RDS and refine clinical procedures.

## Methods

### Overview

This review aims to guide future research in neonatal respiratory care and enhance clinical practice by identifying potential dangers and successful interventions.

### Eligibility Criteria

The eligibility criteria was assessed by PICO (Population, Intervention, Comparison, and Outcome) framework.

For the Population component, we will only accept studies that involve human neonates (≤28 days old) who have been diagnosed with NRDS either clinically or radiographically.

For the Intervention component, infants who have had physical therapy treatments will be included. (1) The interventions will be grouped as active techniques (eg, expiratory flow increase, percussion, and vibration) versus passive or supportive strategies (eg, positioning). Techniques used in chest physical therapy include manual chest compressions, percussion, vibration, and postural drainage. (2) Methods for clearing the airways will include high-frequency oscillation of the chest wall, cough stimulation, or suctioning. (3) Breathing exercises will include methods to enhance lung expansion, include inspiratory training or mild inflation therapy. (4) Positioning therapy will include methods to improve respiratory mechanics and oxygenation, such as lateral tilt or prone positioning. (5) Other treatment will include an expiratory flow increase technique, which is a thoracoadominal movement performed manually by the physiotherapist to clear airway obstructions [6,10].

For the Comparison (C) component, the inclusion of a control group without treatment is necessary in studies, and this group may consist of participants receiving a placebo.. Normal treatment without physiotherapy will include standard medical care for neonates with NRDS, including surfactant replacement therapy. Mechanical ventilation, such as invasive ventilation or CPAP, will be given. Supportive treatment, such as controlling body temperature and fluid intake, will be provided. Different approaches to intervention include other nonphysiotherapy therapies, such as medication or surgery, that target respiratory function will be administered to neonates with NRDS.

In terms of outcome measures, oxygen saturation levels (SpO₂), ABG analysis (eg, PaO₂, PaCO₂, and pH), respiratory rate (breaths per minute), and lung compliance (static or dynamic) will all be used to quantify respiratory function. Validated scoring methods, such as the Downes or Silverman-Andersen scores, will be used to assess the degree of respiratory distress reduction. Length of hospital stay will be monitored. The length of respiratory support, including mechanical ventilation, CPAP, or oxygen therapy, will be documented from the start of the intervention to the end of respiratory support.

For study design, both observational studies (prospective and retrospective cohort studies and case-control studies) and randomized controlled trials (RCTs) that assess the impact of physiotherapy therapies in neonates with NRDS will be included in this review. Regarding language, only English-language research or studies with available translations will be deemed eligible for this study due to translation restrictions (due to resource constraints for translation). Studies published from 2000 to 2025 will be considered for inclusion. Both published article and gray literature will be considered as eligible publication types.

### Information Sources

To find papers that are pertinent to our study issue, we will use both manual and electronic searches. A total of 4 databases—PubMed, Embase, CINAHL, and the Cochrane Library—will be searched electronically (from 2000 to 2024 December). Although it is acknowledged as a search engine rather than a formal database, Google Scholar will be used to supplement the search. Depending on the needs of each database, our search algorithms will blend free texts and MeSH (Medical Subject Headings). Due to the low quality of the evidence, individual case reports, case series, and expert views will not be included in the data extraction process. Papers that are not published or in full text will also not be included.

### Search Strategy

The Cochrane Library, PubMed, Embase, and CINAHL will be thoroughly searched. Physiotherapy will be used, along with MeSH and pertinent keywords such as “Infantile Respiratory Distress Syndrome,” “Neonatal Respiratory Distress Syndrome,” “Respiratory Distress Syndrome, Infant,” “Respiratory Distress Syndrome,” “physiotherapy,” “respiratory function,” “neonates,” “respiratory distress syndrome,” and “New-born” combined with Boolean operators AND/OR. To make sure the most recent research is included, searches will be conducted again prior to the final analysis. In addition, trial registers, investigator contacts, and conference proceedings will be searched for unpublished studies. ProQuest Dissertations & Theses Global and Google Scholar will be used to consult the gray literature. Medline and the Cochrane Library will use MeSH phrases to help find other relevant publications. For different suffixes, truncation (*) and Boolean operators (OR, AND) are used (S1 supplementary file).

### Selection and Data Collection Processes

**Figure 1.**
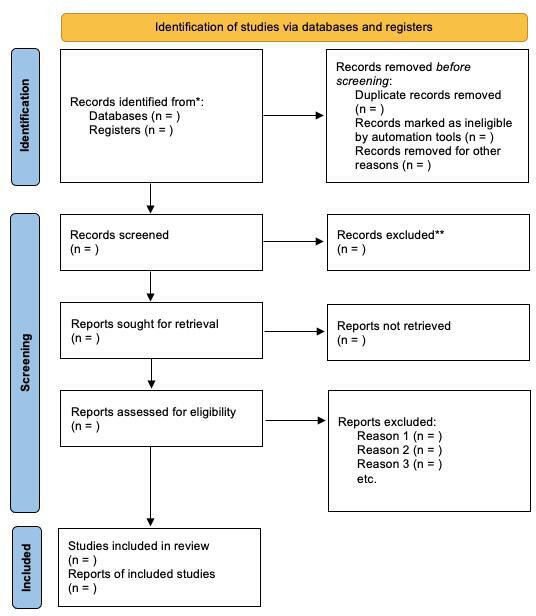
PRISMA-P (Preferred Reporting Items for Systematic Reviews and Meta-Analyses Protocols) flowchart.

SH and IQ are 2 calibrated and independent reviewers and will conduct the study in 2 stages. The first stage involves screening abstracts and titles for alignment with the inclusion criteria and research question. To decide on the final inclusion, the full texts of possibly pertinent research are examined in the second stage. This procedure will be guided by the inclusion and exclusion criteria. In phase 1, the titles and abstracts of the chosen papers will be read by the 2 reviewers, SH and IQ. The entire texts of the previously included papers will be read by the same reviewers (SH and IQ) in phase 2. The selection criteria will be the same as those from the first round. A dual-reviewer approach will be used, and any disagreements will be resolved through consensus discussions or by involving a third independent reviewer (HP). A summary of the study selection procedure is given in Figure 1. This process is now explicitly detailed to ensure consistency and transparency during study selection.

### Data Items

Participant characteristics will include baseline characteristics such as gestational age (mean age and age range), gender distribution, and inclusion and exclusion criteria, respiratory function, and degree of respiratory distress reduction among other pertinent demographic or clinical data. Details of the intervention will include the type of physiotherapy intervention (eg, chest physiotherapy and positioning), its duration, frequency, intensity, and mode of delivery. Information about the sort of control or comparative interventions (such as a placebo, standard care, or no intervention), as well as its length, frequency, and intensity, will be gathered for comparators. Respiratory function will be the main focus of outcome measures. Downes or Silverman-Andersen scores, respiratory rate (breaths per minute), lung compliance (static or dynamic), SpO₂, and ABG analysis (eg, PaO₂, PaCO₂, and pH) will be used to evaluate the extent of respiratory distress reduction and any adverse effects reported. The study design (such as an observational study or RCT), sample size, funding sources to evaluate potential bias, and any conflicts of interest disclosed by the study authors are all examples of study features. To ensure consistent and accurate data extraction and analysis across all included research, preplanned data assumptions and simplifications will be used when needed [11,12].

### Outcomes

Improvements in respiratory function and a decrease in the intensity of respiratory distress are the main goals of this review. SpO₂, ABG analysis (eg, PaO₂, PaCO₂, and pH), respiratory rate (breaths per minute), and lung compliance (static or dynamic) will all be used to quantify respiratory function. These measures will be evaluated using pulmonary function tests or ventilator settings. Data will be collected at preintervention (baseline), postintervention, and at follow-up periods (eg, 24-72 hours and 1 week). Validated scoring methods, such as the Downes or Silverman-Andersen scores, will be used to assess the degree of respiratory distress reduction. The length of hospital stay and the duration of respiratory assistance are examples of secondary outcomes. The length of respiratory support, including mechanical ventilation, CPAP, or oxygen therapy, will be documented from the start of the intervention to the end of respiratory support. This duration will be expressed in hours or days. The number of days from admission to discharge or transfer, recorded at the time of discharge, will be used to calculate the length of hospital stay. These results will be useful in evaluating the immediate and long-term effects of physical therapy treatments on newborns with NRDS.

### Assessment of Risk of Bias in Included Studies

Using proven instruments based on study design, 2 reviewers, SH and IQ will independently evaluate the risk of bias. We will use the Cochrane Risk of Bias 2.0 tool for RCTs and the Risk Of Bias in Non-randomized Studies-of Interventions (ROBINS-I) tool for observational studies. Any disagreements will be settled by consensus with a third reviewer (RR). The weighting of the evidence during synthesis and interpretation will be influenced by the quality assessment’s findings. The authors will discuss the tool and establish the settings for its assessment before applying it. A calibration exercise among the evaluators will also be conducted. Each tool’s suggested presentation of the results will be used. The Robvis website will be used to make figures [13]. If there are enough studies (typically more than 10) to address publication bias, a funnel plot will be used. We will use either Egger or Begg test to statistically analyze the funnel plot’s asymmetry [11,12]. We will perform subgroup and sensitivity analyses, and outline procedures for assessing interrater reliability during bias evaluation. These additions further strengthen our methodological framework.

### Data Synthesis

During synthesis, a hierarchical strategy will be used. Priority will be given to RCT evidence when analyzing and interpreting results. In order to address areas where RCT data are scarce or unavailable, highlight new patterns, or offer supporting evidence, observational studies will be used. When applicable, findings will be categorized by study design to differentiate between data from higher and lower quality sources. Standardized mean differences or relative risks with 95% CIs will be used to measure the effectiveness of treatment for NRDS in order to assess primary outcomes such as respiratory function and a reduction in the severity of respiratory distress. A narrative approach will be the main method used for data synthesis due to the expected heterogeneity among research. Key outcomes, types of interventions, and their reported benefits on respiratory function and the degree of respiratory distress reduction will be highlighted in a descriptive summary of the findings from the included studies. To improve clarity, pictorial representations will be added to a narrative summary. To evaluate variations in the effects of the intervention across participant demographics or intervention attributes, subgroup analyses will be carried out. We will perform subgroup analyses based on intervention type, gestational age, or setting (eg, neonatal intensive care unit vs step-down units), apply random-effects models for meta-analyses, and conduct narrative synthesis when heterogeneity will be too high for pooling [10,11].

### Confidence in Cumulative Evidence

This systematic review will be assessed using the GRADE (Grading of Recommendations, Assessment, Development, and Evaluation) method. In the management of neonates with RDS, the GRADE approach may facilitate a comprehensive evaluation of the evidence supporting the efficacy of physiotherapy treatments.

### Ethical Considerations

This study is a protocol for a systematic review and does not involve the collection of primary data from human participants or animals. Therefore, ethical approval and informed consent were not required. All data to be analyzed will be obtained from previously published studies that had already received ethical approval and informed consent from their respective authors and institutions.

## Results

The time frame for completing this investigation is January 2025-December 2027. The 2 reviewers will be blinded at each step of the process. Regular team meetings will be held to settle disputes or conflicts between the reviewers. This will contribute to increasing the transparency of the evaluation process. Every discussion will be recorded. As soon as the systematic review is complete, it will be submitted for publication. The intended review will be conducted in accordance with guidelines mentioned in the PRISMA-P (Preferred Reporting Items for Systematic Reviews and Meta-Analyses Protocols) checklist. This study has been registered with PROSPERO (CRD42025635782)and PRISMA-P flowchart has been elicited in Figure 1.

## Discussion

### Anticipated Findings

In order to improve respiratory function and decrease respiratory distress in newborns with RDS, this systematic review methodology attempts to thoroughly assess physiotherapy therapies. Numerous physiotherapy strategies, such as posture interventions, respiratory muscle training, airway clearance techniques, and chest physiotherapy, will be methodically examined in this study. Clinical recovery parameters, oxygen saturation levels, mechanical ventilation time, and respiratory function indicators will be the main emphasis of the outcome measures. The methodology includes conducting a thorough literature search, defining precise inclusion and exclusion criteria, evaluating the caliber of studies, and, when appropriate, performing a meta-analysis. The lack of high-quality neonatal physiotherapy studies, possible diversity in therapeutic protocols, ethical research limitations, and the requirement for standardized evaluation techniques are some of the difficulties that researchers foresee. The ultimate objective of the protocol is to offer evidence-based perspectives on the efficacy of physiotherapeutic methods for treating respiratory distress.

### Strengths and Limitations

The Meta-Analyses and Systematic Reviews of Observational Studies (MOOSE) standards and the PRISMA-P were used in the design of this study. The 2 independent reviewers will choose the studies, and a third reviewer will facilitate any disputes over data extraction and study inclusion. The literature will only contain English-only texts due to translation restrictions, which will decrease the total amount of data that may be extracted. In addition to outlining any knowledge gaps, the study will serve as a direction for future research into these areas if there was not enough data available for extraction and analysis. Although there may be a significant chance of bias due to the inclusion of nonrandomized studies, this will be addressed as a restriction in our systematic review and provide valuable information for future work. This systematic review is expected to demonstrate that physiotherapy interventions, including respiratory muscle training, chest physiotherapy, airway clearance techniques, and positioning, improve respiratory function and lessen respiratory distress in neonates with RDS. Our hypothesis is that these interventions will be linked to better overall respiratory outcomes, higher oxygen saturation levels, shorter periods of mechanical ventilation, and superior clinical recovery measures. Depending on data availability and homogeneity, this may involve a meta-analysis in addition to a comprehensive literature search, well-defined inclusion and exclusion criteria, and a quality assessment of chosen studies.

### Conclusions and Direction for Dissemination

In neonates with RDS, the systematic review may offer important new information about the safety and effectiveness of physical therapy therapies for enhancing respiratory function and lowering discomfort. The results could help update neonatal care guidelines and best practices, making sure that therapies are safe and effective for this susceptible group. The findings of this review will be essential for sharing with medical professionals via peer-reviewed journals, clinical conference presentations, and incorporation into university curricula. Furthermore, presenting the results in easily comprehensible formats such as policy briefs, infographics, and webinars can involve a wider range of stakeholders, such as advocacy organizations and health care policy makers. This comprehensive strategy will guarantee that the information reaches every segment of the health care system, impacting clinical practice as well.

### Dissemination Plan

The dissemination plan is listed as follows:

1. Academic dissemination: This study will be presented at conferences for physiotherapy and newborn care and also be published in high-impact journals. Creating policies or suggestions for newborn health care professionals is known as clinical implementation.
2. Public and policy engagement: working together with health care groups to make sure that results influence policy choices pertaining to neonatal care.
3. Open access and media outreach: to increase the systematic review’s reach, make it publicly available and use press releases or social media sites.

## Supplementary material

10.2196/71854 Checklist 1 PRISMA-P checklist.

## Abbreviations

ABG: arterial blood gas
CPAP: continuous positive airway pressure
GRADE: Grading of Recommendations, Assessment, Development, and Evaluation
MeSH: Medical Subject Headings
MOOSE: Meta-Analyses and Systematic Reviews of Observational Studies
NRDS: neonatal respiratory distress syndrome
PaCO_2_: partial pressure of carbon dioxide
PaO_2_: partial pressure of oxygen
PICO: Population, Intervention, Comparison, Outcome
PRISMA-P: Preferred Reporting Items for Systematic Reviews and Meta-Analyses Protocols
RCT: randomized controlled trial
RDS: respiratory distress syndrome
ROBINS-I: Risk Of Bias in Non-randomized Studies-of Interventions
SpO₂: oxygen saturation levels

## References

[1] Sweet DG, Carnielli VP, Greisen G (2023). European consensus guidelines on the management of respiratory distress syndrome: 2022 update. Neonatology.

[2] Bick U, Müller-Leisse C, Tröger J (1992). Therapeutic use of surfactant in neonatal respiratory distress syndrome. Correlation between pulmonary X-ray changes and clinical data. Pediatr Radiol.

[3] Jobe AH (1993). Pulmonary surfactant therapy. N Engl J Med.

[4] Shin JE, Yoon SJ, Lim J (2020). Pulmonary surfactant replacement therapy for respiratory distress syndrome in neonates: a nationwide epidemiological study in Korea. J Korean Med Sci.

[5] Gregory GA, Kitterman JA, Phibbs RH, Tooley WH, Hamilton WK (1971). Treatment of the idiopathic respiratory-distress syndrome with continuous positive airway pressure. N Engl J Med.

[6] Igual Blasco A, Piñero Peñalver J, Fernández-Rego FJ, Torró-Ferrero G, Pérez-López J (2023). Effects of chest physiotherapy in preterm infants with respiratory distress syndrome: a systematic review. Healthcare (Basel).

[7] Ekhaguere OA, Okonkwo IR, Batra M, Hedstrom AB (2022). Respiratory distress syndrome management in resource limited settings-current evidence and opportunities in 2022. Front Pediatr.

[8] Sharath HV, Qureshi MI, Raghuveer R, Saklecha A, Nadipena PT (2024). The effect of physical rehabilitation on oro-motor stimulation, manual airway clearance, positioning, and tactile stimulation (PROMPT) on neonates with respiratory distress syndrome. Cureus.

[9] Xu H, Xu P (2021). Efficacy analysis of different pulmonary surfactants in premature infants with respiratory distress syndrome. Zhonghua Wei Zhong Bing Ji Jiu Yi Xue.

[10] Higgins JPT (2019). Cochrane Handbook for Systematic Reviews of Interventions Version 60.

[11] Almeida CCB, Ribeiro JD, Almeida-Júnior AA, Zeferino AMB (2005). Effect of expiratory flow increase technique on pulmonary function of infants on mechanical ventilation. Physiother Res Int.

[12] Munn Z, Stern C, Aromataris E, Lockwood C, Jordan Z (2018). What kind of systematic review should I conduct? A proposed typology and guidance for systematic reviewers in the medical and health sciences. BMC Med Res Methodol.

[13] Robvis (visualization tool). riskofbias.info.

